# Response of Three *Miscanthus × giganteus* Cultivars to Toxic Elements Stress: Part 1, Plant Defence Mechanisms

**DOI:** 10.3390/plants10102035

**Published:** 2021-09-28

**Authors:** Karim Suhail Al Souki, Clarisse Liné, Francis Douay, Bertrand Pourrut

**Affiliations:** 1 Department of Environmental Chemistry and Technology, Faculty of Environment, Jan Evangelista Purkyně University in Ústí nad Labem, Pasteurova 3632/15, 400 96 Ústí nad Labem, Czech Republic; karim.souki@ujep.cz; 2 Laboratoire Génie Civil et Géo-Environnement (LGCgE), ISA Lille, Junia, 48 Boulevard Vauban, 59046 Lille, France; clarisse.line@ensat.fr (C.L.); francis.douay@junia.fr (F.D.); 3 Laboratoire Écologie Fonctionnelle et Environnement (ECOLAB), Université de Toulouse, CNRS, INPT, UPS-ENSAT (École Nationale Supérieure Agronomique de Toulouse), Avenue de l’Agrobiopôle, 31326 Castanet-Tolosan, France

**Keywords:** *Miscanthus × giganteus*, toxic elements (TE), photosynthetic pigments, secondary metabolites, antioxidant enzymes

## Abstract

*Miscanthus × giganteus* demonstrated good phytostabilization potentials in toxic element (TE) contaminated soils. However, information about its tolerance to elevated concentrations is still scarce. Therefore, an ex-situ pot experiment was launched using three cultivars (termed B, U, and A) grown in soils with a gradient Cd, Pb and Zn concentrations. Control plants were also cultivated in non-contaminated soil. Results show that the number of tillers per plant, stem diameter as well as leaf photosynthetic pigments (chlorophyll *a*, *b* and carotenoids) were negatively impacted by soil contamination. On the other hand, phenolic compounds, flavonoids, tannins, and anthocyanins levels along with the antioxidant enzymatic activities of superoxide dismutase, ascorbate peroxidase and glutathione reductase increased in the plants grown on contaminated soils. Altogether, these data demonstrate that miscanthus is impacted by concentrations of toxic elements yet is able to tolerate high levels of soil contamination. These results may contribute to clarifying the miscanthus tolerance strategy against high contamination levels and its efficiency in phytoremediation.

## 1. Introduction

Human activities (industrial activities, mining, combustion of fossil fuels, waste disposal, agriculture, etc.) are the main sources of toxic element (TE) pollution in soils which may cause critical environmental problems expressed by diverse toxic effects on human health, plants, animals, and microorganisms [1,2].

Remediating polluted sites is a great challenge mainly when contaminants are miscellaneous, persistent, and widespread. Phytoremediation is an environmentally friendly and cost-effective technique that uses plants to reclaim polluted areas by reducing pollutant diffusion and hazards or rendering them harmless [3,4]. Among several plants used in phytoremediation, the promising bio-energy crop *Miscanthus × giganteus* is gaining increased interest. It is recognized for its capacity to diminish human and environmental risks by accumulating TEs in the roots/rhizosphere and limiting their transfer and bioavailability [3]. Besides its carbon sequestrations capacities [4], the plant has demonstrated potential to stimulate bacterial PAH degradation [5,6,7], which can be useful in managing multi-contaminated sites. These capacities, combined with high biomass yield and a remarkable adaptability to different environments, render this C_4_ perennial, rhizomatous, and non-invasive grass a good candidate for phytomanagement of various contaminated areas.

In northern France, around the former lead smelter Metaleurop Nord, miscanthus plants grown on highly contaminated agricultural plots exhibited good development and low element accumulation in its aboveground parts [8,9]. Pelfrêne et al. [10] demonstrated its substantial positive impacts on metal redistribution in soils by decreasing their proportions in the exchangeable fractions, and thus decreasing their oral accessibility to humans. Finally, Al Souki et al. [7,11] highlighted the positive impact of *Miscanthus × giganteus* in restoring the contaminated soil functionality via enhancement of its microbiological and physicochemical parameters.

In general, when exposed to high levels of TEs, plants are negatively impacted. For instance, photosynthesis is impeded by the damage caused to the photosynthetic apparatus, including the inhibition of photosystem II (PSII) and the enzymatic activities implicated in carbon fixation [12]. The decline in amounts of the photosynthetic pigments Chlorophyll *a*, *b* (Chl *a*, *b*) and carotenoids (car) is another sign of the noxious effects on plants of TEs. This may happen either by inhibiting the activity of the enzymes involved in their biosynthesis or as a result of oxidative damage [13]. Secondary metabolites contribute to certain toxins, natural colours, tastes or even odours in plants [14], and play an essential role in defining the interactions these plants have with their surroundings and against different types of stress, including TEs. As a matter of fact, increased amounts, or accumulation, of secondary metabolites is an indication of a defence mechanism that occurs when the plant is subjected to various stress types [15]. Another sign of plant TE oxidative stress is the excess production of reactive oxygen species (ROS) such as superoxide (O_2_^.−^), H_2_O_2_ and hydroxyl radical (^.^OH) [16]. Such an excess can cause severe deleterious oxidation to the majority of cell components. However, plants have developed certain ROS scavenging and detoxification mechanisms including enzymatic and non-enzymatic antioxidants. For instance, plants activate certain antioxidant enzymes such as superoxide dismutase (SOD), ascorbate peroxidase (APX), catalase (CAT), glutathione reductase (GR) and dehydroascorbate reductase (DHAR) and/or non-enzymatic antioxidants such as the photosynthetic pigment (car), ascorbic acid, or some secondary metabolites such as the phenolic compounds (flavonoids, anthocyanins, tannins, lignins, phenolic acid and other related compounds) [15].

Within this context, investigations and research papers regarding the pollutant’s effects on miscanthus and its ability to tolerate potential stress are still scarce. The main objective of the current study was to determine the effects of cultivating *Miscanthus × giganteus* in the polluted soils surrounding the former Pb smelter Metaleurop Nord, with a gradient concentration of Cd, Pb, and Zn. For this purpose, we studied the impacts on three different miscanthus cultivars via: (i) plant growth parameters and Cd, Pb, and Zn concentrations in the leaves, (ii) photosynthetic pigment (chlorophyll *a*, chlorophyll *b*, and carotenoids) and secondary metabolites levels (phenolic compounds, tannins, flavonoids, and anthocyanins), and (iii) antioxidant enzymatic activities in the leaves (superoxide dismutase (SOD), ascorbate peroxidase (APX), and glutathione reductase (GR)). 

## 2. Materials and Methods

### 2.1. Soil and Plannt Origins/Preparations

The area surrounding the former lead smelter Metaleurop Nord is heavily contaminated by Cd, Pb, and Zn [17]. Soil samples (plowed horizon, 0–25 cm) were collected from agricultural plots termed M200, M500, M750, and M900, corresponding to their approximate Pb concentration (in mg kg^−1^ of soil), which decreases as the distance from the smelter increases [18]. M200 (50°24′52″ N, 3°01′51″ E, Courcelles-les-Lens) and M500 (50°25′49″ N, 3°02′13″ E, Evin-Malmaison) plots are 1.8 km southeast and 1.4 km northeast of the smelter. Meanwhile, M750 and M900 soils were collected from a plot in Evin-Malmaison (50°26′15.0″ N 3°01′05.7″ E), approximately 1 km away from Metaleurop Nord. Moreover, to verify the contamination influence, uncontaminated soil was added. Samples were collected from a control agricultural field termed MC, which is 75 km from the smelter (50°20′46″ N 2°12′15″ E). All the soil samples were homogenized, dried, and sieved through a 10-mm mesh. 

Three different cultivars of *Miscanthus × giganteus* (referred to as B, U, and A) were used throughout the experiment. B and A miscanthus were supplied by NovaBiom (Champhol, France) and Rhizosfer (Brienne sur Aisne, France), and the U one was provided from Iowa State University (Iowa, USA). Rhizomes were cut into small pieces, one fragment (5–7 cm long, 2–3 buds) of which was grown in polyethylene pots (9 × 9 × 9 cm) filled with potting compost, until obtaining miscanthus plantlets (20–25 cm in height). The planted compost was kept wet by constant watering.

### 2.2. Experimental Design

For a period of 18 months, an *ex situ* experiment was conducted in May, in an area away from roads within Lille 1 University campus (50.6090° N, 3.1381° E). 100 kg from the collected homogenized soils (MC, M200, M500, M750, and M900) were equally divided in five pots (light grey in colour to avoid temperature elevation). Miscanthus plantlets (B, U, and A) were transferred from the small pots to the 20-kg soil pots (two plantlets per pot).

In total, 75 planted pots were used (3 different miscanthus cultivars (B, U and A) × 5 soils (MC, M200, M500, M750 and M900) × 5 replicates (soil treatment and miscanthus cultivar)). The pots were placed over wooden rafters to avoid contact with the ground and randomly distributed over the location to avoid point and borderline effects. In addition to rainwater, soil humidity was maintained by regular watering throughout the entire experiment (further data concerning the experimental plot and design as well as growth conditions can be found in Supplementary Materials, Figure S1). Weeds were manually removed and left on the soils surface to avoid element exportation.

Finally, it is noteworthy to mention that there are other possible sources of stress factors that might impact the plants such as insects, pathogens etc., however, all existing papers indicated that humidity (which was handled in the current experiment) or soil pollution are the sole sources. Moreover, the uncontaminated samples included in this study could have given indications of other stress sources, as a result the current work focuses only on the impact of TEs on the health of the miscanthus. 

### 2.3. Soil Sampling and Analysis

Once the experimental site was set up, fresh composite soil samples were collected from the pots using an auger, dried at 40 °C for 24 h, ground and sieved to 2 mm and 250 µm. All soil analyses were conducted following standardized protocols.

Soil particle-size distribution was determined through sedimentation and sieving upon organic matter removal by H_2_O_2_ following the NFX 31-107 French standard. pH (H_2_O) was determined according to ISO 10390 standard by stirring a mixture of soil and deionized water (1:5, *v*/*v*).

For determining Cd, Pb, and Zn pseudototal concentrations, soil acid digestion was performed according to Al Souki et al. [3] with the use of a digestion plate (HotBlock^TM^ Environmental Express, Charleston, SC, USA). An aqua regia solution (HCl + HNO_3_, 3:1, 6 mL) was added and the aliquot was heated at 120 °C for 120 min. The extraction and analysis quality control was provided by introducing two internal reference samples and a certified soil reference (CRM 141, IRMM, Geel, Belgium). Cd, Pb and Zn concentrations in the extracts were detected by atomic absorption spectrophotometry (AA-6800, Shimadzu, Kyoto, Japan).

### 2.4. Plant Sampling and Analysis

#### 2.4.1. Plant Growth Parameter Measurements, Leaf Sampling and Preparation

In October, by the end of the first growing season (5 months of cultivation), plant growth parameters (stem height and diameter) were measured. Number of tillers per plant was counted as well. Total aerial biomass was not harvested and measured, in order not to prevent nutrient translocation towards the rhizomes, and thus not to compromise the 2nd year of the experiment. 

To assess plant health, three leaves (4th, 5th, and 6th foliar stage) were harvested from each pot and immediately flash frozen in liquid nitrogen. Samples were then conserved at −80 °C until biomarker analysis. 

The other leaves were also harvested to measure TE concentrations, put into plastic bags, and kept in a coolbox. In the laboratory, leaves were washed three times with osmosed water to remove dust particles and then oven-dried at 40 °C for 48 h. Afterwards, samples were ground into fine powder using a knife mill (GM200, Retsch, Haan, Germany) before analysis.

#### 2.4.2. Toxic Element (TEs) Concentrations in Leaves

To determine the leaf Cd, Pb and Zn concentrations, 300 mg of ground leaf powder was acid digested with HNO_3_ (70%) and heated at 95 °C for 75 min, followed by adding H_2_O_2_ (30%) and another 180 min heating prior to the addition of osmosed water. Finally, concentrations in the extracts were determined through atomic absorption spectrophotometry (AA-6800, Shimadzu, Kyoto, Japan). Quality control for chemical extraction and digestion was performed by including blanks, internal, and certified (Polish Virginia tobacco leaves, INCTPVTL-6, Warsaw, Poland) reference materials [3].

#### 2.4.3. Antioxidant Enzymatic Activities

Antioxidant enzymatic activity assays were evaluated spectrophotometrically using a plate reader (Thermo Scientific Multiskan™ GO, Illkirch-Graffenstaden, France). In brief, five foliar discs (0.5 cm in diameter) per plant sample were collected from the frozen leaves using a manual punch. They were then placed in a 96-deepwell plate (2 mL) with one 4-mm-diameter glass bead. Samples were thereafter ground in frozen conditions with a Mixer Mill MM 400 (Retsch, Haan, Germany), twice for 1.5 min at 30 Hz, after adding 1 mL of ice-cold Tris extraction buffer pH 7.0 containing 0.01 M EDTA, 0.4 M PVP, 0.05 ascorbate, 11.44 mM β-mercaptoethanol, and protease cocktail inhibitor. Samples were homogenized at 15 Hz for 2 minutes with the MM400 grinder. Afterwards, plates were centrifuged at 5000× *g* for 15 min at 4 °C. Finally, supernatants were collected and protein content was determined according to Bradford [19], using bovine serum albumin (BSA, Sigma, Saint-Quentin-Fallavier, France) as standard.

Total superoxide dismutase (SOD) activity was estimated according to its capacity to inhibit nitro blue tetrazolium (NBT) reduction, and ascorbate peroxidase (APX) was determined by the absorbance decline at 290 nm as a result of ascorbate oxidation [3]. Glutathione reductase (GR) was detected as the decrease in absorbance at 340 nm due to NADPH oxidation. The assay is based on the reduction of oxidized glutathione (GSSG) by NADPH in the presence of GR [20].

#### 2.4.4. Secondary Metabolism Molecule and Photosynthetic Pigment Quantification

Photosynthetic pigments, phenolic compounds, tannins, flavonoids, and anthocyanins were evaluated spectrophotometrically using a plate reader (Thermo Scientific Multiskan™ GO). In brief, two foliar discs (0.5 cm in diameter) per plant sample were collected from the frozen leaves using a manual punch. Then they were placed in a 96-deepwell plate (2 mL) with one 4-mm-diameter glass bead. Samples were thereafter ground in frozen conditions using a Mixer Mill MM 400 (Retsch), twice for 1.5 min at 30 Hz. After the addition of 1.5 mL of ice-cold 95% methanol in each well, samples were homogenized at 15 Hz with the MM400 grinder for a period of 2 min. Finally, the plates were left in the dark for 24 h and 48 h of incubation. 

After 24 h of incubation, photosynthetic pigment (Chl *a*, *b* and car) concentrations were calculated according to Al Souki et al. [3] after measuring the solution’s absorbance at 470, 652, and 666 nm. 

After 48 h of incubation, plates were centrifuged at 5000× *g* for 5 min, prior to secondary metabolism molecule extraction. Total phenolic compounds were determined based on Folin Ciocalteu assay. In details, the 200-µL reaction mixture contained 20 µL of supernatant, 40 µL of Folin reagents (10% *v*/*v*), and 0.098 mM of Na_2_CO_3_. The mixture was allowed to stand 2 h at room temperature for colour development and then absorbance was measured at 510 nm. Concentrations of phenolic compounds were calculated using a standard curve of gallic acid. Results were expressed as mM of gallic acid equivalent (GE) per gram of fresh leaf weight. Flavonoid content was determined using the aluminium chloride method with catechin as the reference compound. A reaction mixture was prepared with 25 µL methanolic extract, 0.00724 mM NaNO_2_, 0.01125 mM AlCl_3_, and 0.05 mM NaOH. The mixture was homogenized for 1 min and absorbance was measured at 595 nm. Flavonoid concentrations were calculated using a standard curve of catechin. The results were expressed as mg catechin equivalent (CE) per gram of fresh weight of leaf. For tannins, the reaction mixture contained 50 µL of methanolic extract and 100 µL of vanillin solution 1%. The mixture was left in the dark for 15 min and absorbance was measured at 500 nm. Tannin concentration was calculated based on a standard catechin curve. Results were expressed as mg L^−1^ catechin equivalent (CE) per gram of fresh weight of leaf. Anthocyanins were measured using the differential pH method based on the property of anthocyanin pigments to change colour with pH. Two dilutions of the same sample were prepared, the first one in potassium chloride buffer (0.2 M, pH 1.0) and the second in sodium acetate buffer (0.4 M, pH 4.5). After equilibration at room temperature for 15 min, the absorbance was read at 510 and 700 nm. The results were expressed as mg cyaniding 3-glucoside equivalent per gram of fresh weight of leaf.

### 2.5. Statistical Analysis

Analysis of variance and Fisher test were performed for modalities comparison and significance (*p* ≤ 0.05). Tukey HSD test was used for pair-wise comparisons upon the presence of statistically significant differences. XLSTAT software (Paris, France) was used to perform all the statistical analyses.

## 3. Results

### 3.1. Granulometry, pH and Pseudototal Toxic Element (TE) Concentrations of Soils

Soils used in the experiment were analysed for particle size distribution, pH and pseudototal TE concentrations. Results presented in Table 1 demonstrate that silt was dominant in all the soils ranging from 49.7% in M500 soil to 69.5% in MC soil.

M500 soil was more clayey (30.6%) than the other soils. The pH values in soils fluctuated between slightly acidic in MC (6.4) and slightly basic in M500 (7.6). Soil TE concentration validated the previous data, ranging from 20 to 50 times above the regional agricultural background values (0.42, 38, and 74 mg kg^−1^, corresponding to Cd, Pb, and Zn, respectively). Finally, concentrations in MC coincided with the regional background values.

### 3.2. Plant Growth Parameters and Toxic Elements (TE) Concentration in Leaves

Table 2 presents the Cd, Pb, and Zn accumulation in miscanthus leaves as well as the plant stem diameter and number of tillers at the end of the first growing season. Tiller heights were also measured in each pot. However, during the first growing season, the emergence of tillers was strongly heterogeneous among pots with the same TE concentrations, resulting in very high standard deviation values. Accordingly, results aren’t presented.

Leaf TE concentration increased according to the soil concentrations in which the plants were cultivated. No differences in TE accumulation were observed between the three miscanthus cultivars. In MC soil, Cd concentration in the leaves was below the limit of detection (0.4 mg kg^−1^). Highest Cd leaf concentrations (2.2–2.3 mg kg^−1^) were detected in plants cultivated in the most polluted soil (M900). The same pattern was noted concerning Zn, where leaf concentrations increased from 32.2, 21.6, and 39.0 mg kg^−1^ (corresponding to B, U, and A cultivars, respectively) in MC soil to 74.0, 68.8, and 71.2 mg kg^−1^ in M900. However, Pb concentrations in leaves were always below the limits of detection (4.3 mg kg^−1^) in all the samples.

Significant variations were also revealed concerning plant growth (Table 2). Highest number of tillers was detected in MC soil (8, 14, and 8 tillers per plant, corresponding to B, U, and A cultivars, respectively). Despite the decrease in tiller numbers of B and U cultivars grown in the contaminated soils (approximately four tillers per pot), results did not display significant differences between the modalities. However, U cultivar exhibited significant differences between the uncontaminated and contaminated soils, recording a decrease from 14 tillers in the former to 5 in M900, a 62.9% decrease.

Stems of B and A cultivars planted in the uncontaminated MC soil were thicker than those present in the contaminated pots (8.2 and 7.4 mm respectively). However, significant differences were noted only in B plants cultivated in the contaminated soils. No significant differences were detected in U cultivar, as the stems displayed approximately same thickness in the contaminated and uncontaminated soils (6.1 mm in MC and 6.2 mm in M900).

### 3.3. Antioxidant Enzymatic Activity Assays

Antioxidant enzymatic activities were significantly boosted in miscanthus leaves as a response to the soil TE augmentation.

**Figure 1:**
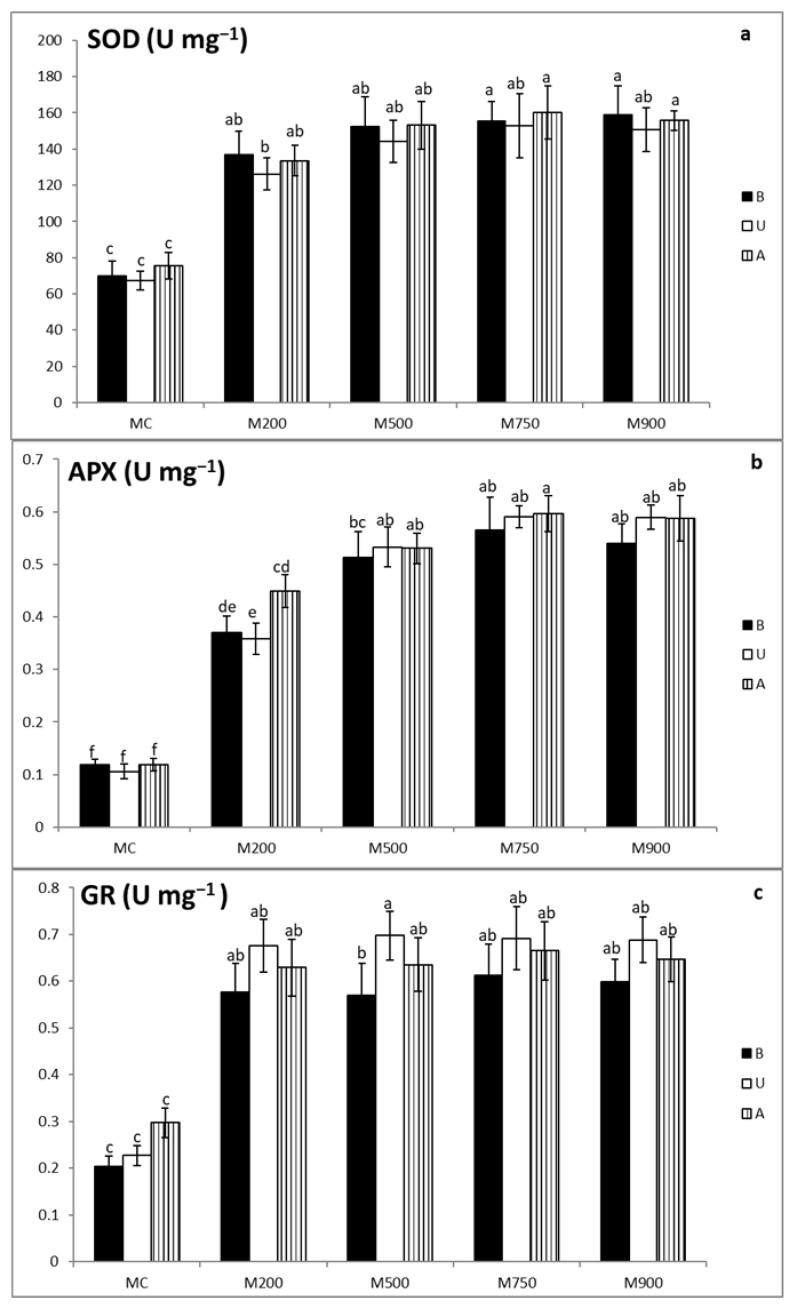
SOD (**a**) APX (**b**) and GR (**c**) activities in the leaves of three different miscanthus cultivars (B, U, and A) cultivated in soils with gradient TE concentrations (MC-control, M200, M500, M750, and M900). Values are presented as means ± SD. Different letters refer to significant differences between plants (Turkey HSD test, *n* = 5, *p* ≤ 0.05).

Lowest superoxide dismutase (SOD) activities (Figure 1a) were observed in plant leaves cultivated in MC soil (69.7, 67.2, and 75.5 U mg^−1^ FW corresponding to the B, U, and A cultivars, respectively) while highest values in those planted in M900 soil for B cultivar (158.8 U mg^−1^ FW) and M750 soil for U and A cultivars (153.0 and 160.2 mg^−1^ FW, respectively). Compared with the uncontaminated leaves, the increase fluctuated between 96.5, 87.7, and 76.8% to 127.7, 127.5 and 112.1 % in B, U, and A cultivars respectively. Significant differences were recorded between plants cultivated in the contaminated and uncontaminated soils. However, they were not detected among contaminated pots.

(Figure 1b) shows that the lowest ascorbate peroxidase (APX) activities were in the leaves of plants cultivated in MC soil (0.1 U mg^−1^ FW in B, U, and A cultivars). Highest values on the other hand were detected in those planted in M750 soil (0.6 U mg^−1^ FW in B, U, and A cultivars). In comparison with the control plants, APX activities in B, U, and A cultivars increased respectively from 212.2, 237.9, and 279.0% in M200 soil, to 376.4, 456.9, and 402.9% in M750 soil. Significant differences were mainly found between the uncontaminated and contaminated soils.

The lowest glutathione reductase (GR) activities (Figure 1c) were demonstrated in the leaves of plants cultivated in MC soil (0.2, 0.2, and 0.3 U mg^−1^ FW corresponding to B, U, and A cultivars, respectively). The most elevated levels were present in those planted in M750 soil for the B and A cultivars (0.6 and 0.7 U mg^−1^ FW, respectively) and in M500 soil for the U cultivar (0.7 mg^−1^ FW). The GR increase in the contaminated B, U and A cultivars ranged respectively between 181.2, 197.7 and 111.8% to 201.9, 207.1 and 123.9%. Significant differences were found between the plants cultivated in uncontaminated and contaminated soils, but not among those present in the contaminated soils.

### 3.4. Secondary Metabolites Quantification

**Figure 2:**
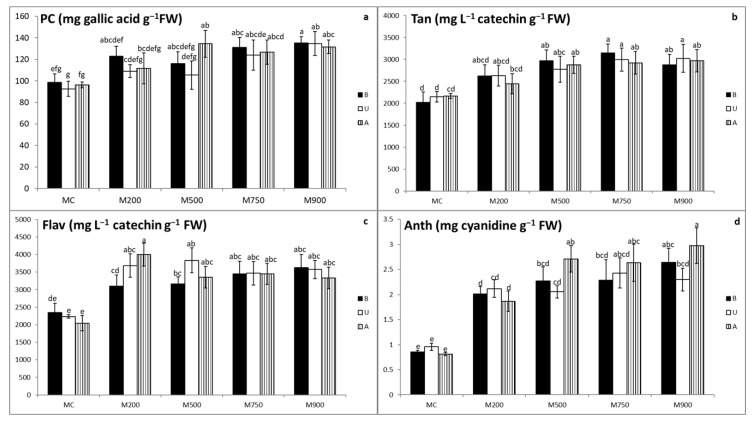
Phenolic compound, PC (**a**), tannin, Tan (**b**), flavonoid, Flav (**c**) and anthocyanin, Anth (**d**) concentrations in the leaves of three different miscanthus cultivars (B, U, and A) cultivated in soils with gradient TE concentration (MC-control, M200, M500, M750, and M900). Values are presented as means ± SD. Different letters refer to significant differences between plants (Turkey HSD test, *n* = 5, *p* ≤ 0.05).

Miscanthus plants cultivated in contaminated soils exhibited increasing phenolic compound, tannin, flavonoid, and anthocyanin concentrations in their leaves compared with those grown in uncontaminated soils (Figure 2a–d).

Phenolic compound concentrations in the control B, U, and A cultivars were 98.6, 92.7, and 96.3 mg gallic acid g^−1^ FW, respectively, representing the lowest quantities among the modalities (Figure 2a). On the other hand, the highest phenolic compound concentrations were recorded in M900 soil for B and U cultivars (135.2 and 134.8 mg gallic acid g^−1^ FW, respectively) and in M500 soil for A cultivar (134.6 mg gallic acid g^−1^ FW). Minimal augmentation was detected in B and U plants cultivated in M500 soil (17.7 and 13.7% respectively) and A cultivar in M200 soil (15.9%), whereas maximal concentration increase was detected in the leaves of B and U plants cultivated in M900 soil (37.1 and 45.4%, respectively), and A plants cultivated in M500 soil (39.7%). Nevertheless, significant differences in the phenolic compounds’ concentrations started to appear in plants cultivated in the highly contaminated pots (M500 and above).

Lowest tannin concentrations (Figure 2b) were recorded in the miscanthus leaves sampled from MC soil (2018.7, 2144.0, and 2168.1 mg L^−1^ catechin g^−1^ FW corresponding to B, U, and A cultivars, respectively), whereas the highest values were observed in the U and A plants of M900 soil (3023.6 and 2970.5 mg L^−1^ catechin g^−1^ FW, respectively) and in M750 soil for B plants (3150.2 mg L^−1^ catechin g^−1^ FW). Concentrations increased from 29.7, 22.8, and 12.9% in B, U, and A cultivars, respectively, grown in M200 soil, to 56.1% in B plants grown in M750 soils and 41.0 and 37.0% in U and A cultivars, respectively, in M900 soil. Significant differences were detected starting from M500 soil.

Flavonoid results (Figure 2c) show that the lowest concentrations were detected in the plants cultivated in MC soil (2347.5, 2235.5, and 2044.8 mg catechin L^−1^ g^−1^ FW in the B, U, and A cultivars, respectively), whereas the highest concentrations were detected in M900 soil for B cultivar, M500 for U cultivar, and M200 for A cultivar (3631.8, 3831.9, and 3997.1 mg catechin L^−1^ g^−1^ FW in the B, U, and A cultivars, respectively). Lowest augmentation was detected in B plants cultivated in M200 soil (32.1%), U plants of the M750 soil (55.1%), and A plants of the M900 soil (62.9%), whereas the highest increases were displayed by the B plants in M900 soil (54.7%), U plants in M500 soil (71.4%), and A plants in M200 soil (95.5%). However, significant differences were mainly detected between the contaminated and uncontaminated soils.

Anthocyanin concentrations (Figure 2d) indicated that the lowest concentrations were measured in the uncontaminated leaves (0.9, 1.0, and 0.8 mg cyanidin g^−1^ FW corresponding to B, U, and A cultivars, respectively), whereas B and A plants in M900 soil recorded the highest values (2.6 and 3.0 mg cyanidin g^−1^ FW, respectively). U plants displayed their highest anthocyanin concentrations in M750 soil (2.4 mg cyanidin g^−1^ FW). Compared with control plants, anthocyanin concentrations in the contaminated B, U and A cultivars increased between 134.0, 114.8 and 129.3%, and reached 207.3, 154.0 and 266.1% respectively. Significant differences were detected between the uncontaminated and contaminated plants. Moreover, these differences existed among the cultivars planted in the contaminated soils as well (B plants cultivated in M900 soil and U cultivar grown in M500, M750, and M900 soils). 

### 3.5. Photosynthetic Pigment Quantification

**Figure 3:**
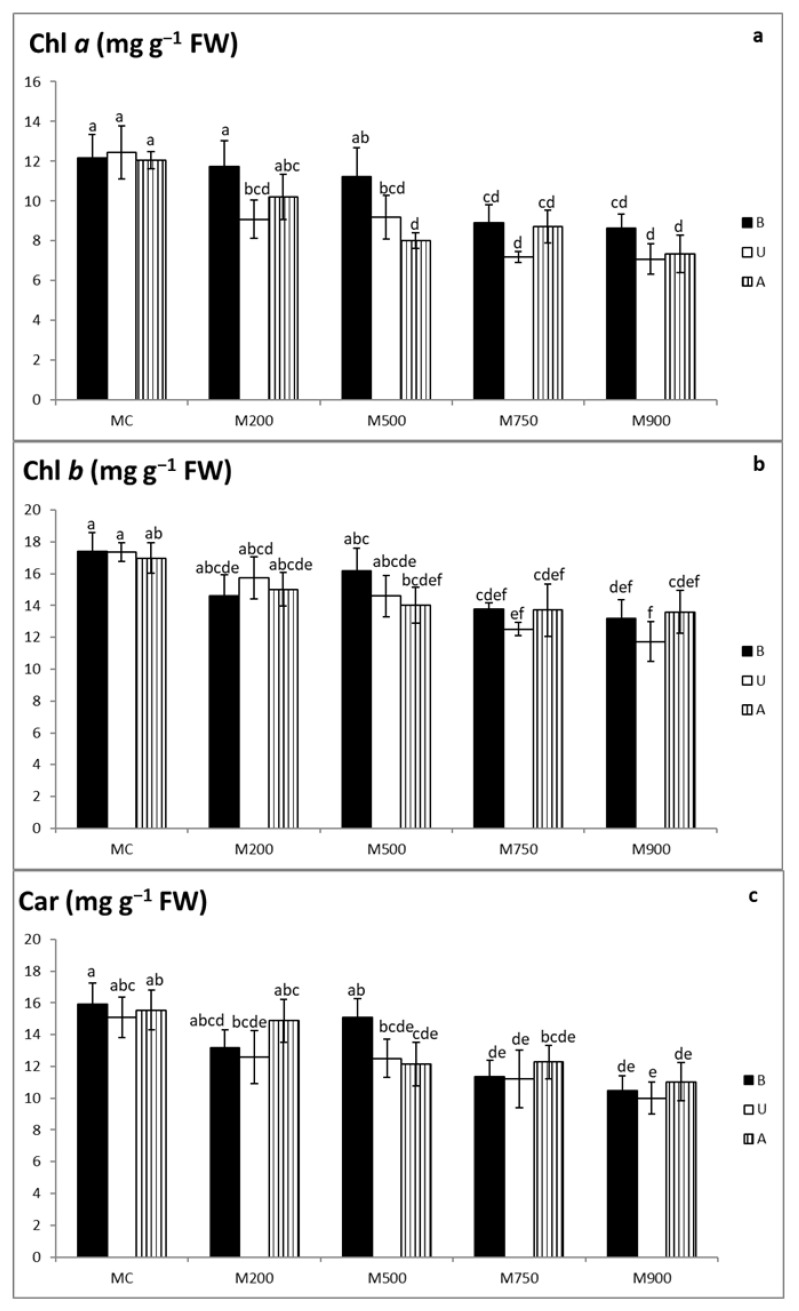
Chlorophyll, Chl *a*, *b* (**a**,**b**) and carotenoid, Car (**c**) contents in the leaves of three different miscanthus cultivars (B, U, and A) cultivated in soils with gradient TE concentration (MC-control, M200, M500, M750, and M900). Values are presented as means ± SD. Different letters refer to significant differences between plants (Turkey HSD test, *n* = 5, *p* ≤ 0.05).

Photosynthetic pigment contents significantly decreased in the three miscanthus cultivars grown in contaminated soils (Figure 3a–c).

Chlorophyll *a* content (Figure 3a) significantly declined from 12.2, 12.5, and 12.1 mg g^−1^ FW corresponding to B, U, and A cultivars, respectively, cultivated in MC soil, to 8.6, 7.1, and 7.3 mg g^−1^ FW in those cultivated in M900 soil. Reductions varied from 3.4, 27.1, and 15.4% in the leaves of B, U, and A cultivars, respectively, in M200 soil, to 29.1, 43.2 and 39.2% in M900 soil. Within the cultivars and in comparison with their corresponding controls, significant variations started to appear in B plants cultivated in M750 soil, U plants grown in M200 soil, and A plants cultivated in M500 soil.

Highest chlorophyll *b* concentrations (Figure 3b) were measured in plants cultivated in MC soil (17.4, 17.4, and 17.0 mg g^−1^ FW corresponding to the B, U, and A cultivars, respectively), whereas the lowest concentrations were observed in plants cultivated in M900 soil (13.2, 11.7, and 13.6 mg g^−1^ FW corresponding to B, U, and A cultivars, respectively). The decrease in B cultivar was between 7.1 and 24.3 % in plants cultivated in the contaminated soils. As for U and A cultivars, the value ranged from 9.3 to 11.6% and 20.0 to 32.4% respectively. Significant differences appeared in the plants cultivated in M750 soil for the three cultivars.

Highest carotenoid (car) contents were recorded in the uncontaminated leaves (15.9, 15.1, and 15.6 mg g^−1^ FW corresponding to B, U, and A cultivars, respectively), whereas the lowest values were obtained in those of M900 soil (10.5, 10.0, and 11.0 mg g^−1^ FW corresponding to B, U, and A cultivars, respectively) (Figure 3c). Compared with car contents in the control leaves, the drop ranged between 5.2% in the leaves of B cultivars cultivated in M500 soil, and 17.2 and 4.3% in the leaves of U and A cultivars, respectively, grown in M200 soil, to 34.3, 33.8, and 29.1% in those of B, U, and A cultivars, respectively, cultivated in M900 soil. Referring to their controls, car concentrations in the leaves of B and U cultivars began to display significant differences in the plants cultivated in M750 soil. A cultivar displayed significant differences in comparison to the controls in the plants cultivated in M500 and M900 soils.

## 4. Discussion

*Miscanthus × giganteus* has demonstrated considerable phytostabilization capacities in different polluted areas. Besides accumulating toxic elements (TEs) mainly in their underground parts, they contribute to restoring soil functionality and biological activities [7,11,21]. However, the distinctive attribute of miscanthus is its ability to thrive and develop well in such degraded areas. Acclimation to stressful conditions prevailing in these areas demands certain adaptations, which result in strong physiological, enzymatic, and molecular alterations.

In the present study, leaf TE concentrations increased in the three cultivars along with the TE augmentation in soil (Table 2). These data agree with other results obtained *in situ* on several contaminated plots surrounding Metaleurop Nord, with gradient soil concentrations [9]. In addition to the experiments in Metaleurop Nord, the current results agree with the outcomes of Guo et al. [22] who demonstrated that under 200 μM Cd treatment (hydroponic solution), Cd concentrations in the leaves of *M. sinensis*, *M. floridulus*, and *M. sacchariflorus* were 146, 210, and 71 μg g^−1^ dry weight, respectively. Andrejić et al. [23], Jiang et al. [24] and Zhang et al. [25] also showed that Zn, Cd/As and Cd concentrations in miscanthus aboveground parts increased proportionally along with the spiked-soil concentrations. The rise in the leaf TE concentrations could be a result of their increased exposure and uptake/translocation due to the limited soil volume and quantity in pots [18,21].

Globally, miscanthus reaction to TE stress in the present study was expressed by significant reduction in the tiller numbers per plant in cultivar “U” (14 tillers in MC soil to 5 tillers in M900 soil), as well as the stem diameter in cultivar “B” (8.2 mm thick in the plants cultivated in MC soil to 4.9 mm in those planted in M900 soil) (Table 2). The results align with the work of Fernando and Oliveira [26] who demonstrated negative effects of metal-contaminated soils on tiller numbers per *M. x giganteus* plant. In the present study, the decline in plant growth may be an outcome of TE impacts on the main metabolic processes such as photosynthetic activities, respiration, as well as nutrient and water uptake [13]. 

Generally, oxidative stress is one of the critical consequences of exposure to high TE concentrations in higher plants. Consequently, reactive oxygen species (ROSs) are produced, including superoxide anion (O_2_^.-^), hydroxyl radical (OH^.^), and hydrogen peroxide (H_2_O_2_), which are strong oxidative agents that might cause irreparable damage to biomolecules such as DNA, proteins, and lipids, thereby affecting plant growth and development, which might eventually lead to cell death [27,28]. However, plants possess a series of antioxidant enzymatic systems involving SODs, peroxidases (including APs), GRs, and others, as well as nonenzymatic constituents including ascorbate, glutathione (GSH), and secondary metabolites that can counteract the negative influence of ROSs, to restore redox homeostasis and normal metabolism [29].

In the current work, the antioxidant enzymes activities SOD, APX, and GR (Figure 1a–c) in the leaves of the three miscanthus cultivars (B, U, and A) displayed a significant accretion in the contaminated soils compared with the control ones (between 100 and 400% in the most contaminated M750 and M900). The data obtained are in total agreement with the results of Guo et al. [18] with *M. sinensis*, *M. floridulus*, and *M. sacchariflorus* exposed to Cd in hydroponic conditions. These authors showed that SOD, APX, as well as GR increased in all three species. It is noteworthy to mention that the increase in the enzymatic activities mentioned is more acute in the results reported by Guo et al. [18] in which the activities augmentation at the highest Cd concentration exceeded 4-fold over those obtained in the controls. Firmin et al. [16] also recorded a 5-fold increase in SOD activities in miscanthus leaves when cultivated in highly contaminated soils.

Secondary metabolites are also important in plant tolerance and ROS detoxification [30]. Our results (Figure 2a–d) clearly demonstrate the increase in phenolic compound, tannin, flavonoid, and anthocyanin concentrations in the three cultivars, mainly those cultivated in the highly contaminated soils (M500, M750, and M900). Various groups of phenolic compounds exist in plants, discriminated by their number of constitutive carbon atoms associated with the structure of the basic phenolic skeleton. These include the simple phenolic compounds, tannins, flavonoids, and anthocyanins. Phenolic compounds possess high capacity to chelate TEs because of the hydroxyl and carboxyl groups existing in their structure [31]. Analogous to all other phenolic compounds, tannins are reported to exhibit antioxidant properties by forming variable affinity complexes with TEs and are thus involved in their detoxification [32]. Flavonoids are considered to be plant metabolites and acquire their antioxidative capacity from their molecular structure, comprising conjugated double bonds in addition to the functional groups in the rings. The main role of flavonoids is lowering the production of and quenching ROSs [33]. Anthocyanins can also be released as a reaction against TE stresses and are believed to boost the antioxidant response of plants in order to sustain their regular physiological status against both biotic and abiotic stresses [34]. Šamec et al. [35] also discussed accretion in the flavonoid concentration following biotic and abiotic stresses, such as wounding, drought, metal toxicity, and nutrient deprivation. Finally, the accumulation of phenolic compounds in leaf tissues was also observed in various species exposed to TEs and in plants grown in different polluted soils [36,37]. Altogether, the increase in phenolic compound concentrations might be a clear indication of their implication in miscanthus protection against TE stress via effective addition of defensive responses to the plant repertoire [31,35].

Another significant indicator of TE-induced stress is the modification of photosynthetic pigments in leaves. Chlorophyll loss is one of the frequent symptoms of TE exposure [37]. Meanwhile, carotenoid production is enhanced or reduced depending on the types of TEs present [34]. Carotenoids are also plant pigments functioning as nonenzymatic antioxidants. They play an influential role in protecting chlorophyll pigments and plant organs under stress conditions by either dissipating excess excitation energy such as heat or scavenging ROSs and restraining lipid peroxidation [34]. In the present study, the results (Figure 3a–c) demonstrate that TE exposure maximally reduced chlorophyll *a* by 29.1, 43.2, and 39.2% in B, U, and A cultivars, respectively. Chlorophyll *b* concentrations were affected as well. Finally, carotenoid contents showed significant reductions in the three miscanthus cultivars as the soil TE concentration increased (maximum 34.3, 33.8, and 29.1% in B, U, and A cultivars, respectively). These results agree with Andrejić et al. [23] and Guo et al. [22], who demonstrated a decrease in the above-mentioned pigments in miscanthus species with the increase in TE contents, reaching a maximum of more than 50% at elevated concentrations, which might be considered the most intense response in comparison with the results obtained in the present study. Zhang et al. [25] also recorded a more than 30% decrease in the photosynthetic pigment contents of *M. sacchariflorus* leaves as Cd concentrations increased in the soils. The diminution in chlorophyll pigments might be linked to the direct effects of TE accumulation on photosynthesis by impairing chlorophyll biosynthesis or the increased TE-induced activity of chlorophyllase [27]. According to Rezayian et al. [38], chloroplasts have a complex system of membranes rich in polyunsaturated fatty acids, which are potential targets for peroxidation. This could also partially explain the deleterious effects of TEs on the chlorophyll contents and other photosynthetic parameters. Nevertheless, it is essential to mention that sometimes the chlorophyll content reduction might be an adaptive defensive response in the leaves of plants grown under contaminated environments, which permits them to protect the photosynthetic apparatus from photo-inhibition and photo-oxidation and thus enduring stressful conditions [39,40]. In addition, the present carotenoids decline may be an outcome of reduced synthesis and/or their enhanced oxidative degradation by the imposed oxidative stress [41]. Finally, according to Baek et al. [34], carotenoids play an important role in protecting against mild TE stress conditions, but with increasing concentrations, anthocyanins participate more effectively in protecting the plant. This might explain the results, in which it is evident that carotenoid levels decreased in the miscanthus plants as TE concentrations increased, while anthocyanin inductions increased with rising contamination.

Altogether, the collective work of the antioxidant enzymatic network and secondary metabolites might lead to an alleviation of the damage caused to the cells through decreasing ROS levels and oxidative damage to lipids [40]. This is considered a good indicator of the miscanthus antioxidant defence system efficiency, which could scavenge ROSs, maintain integrity of the chloroplast, restore redox homeostasis and normal metabolism under stress [22,42], thus confirming their high tolerance to elevated TE concentrations [43].

## 5. Conclusions

The present study focuses on the response of three miscanthus cultivars to increasing TE concentrations (Cd, Pb, and Zn) in soil. The results show that the number of tillers and stem diameter were slightly affected by soil contamination. The inhibitory effects on the photosynthetic pigments were evident when comparing the contaminated and uncontaminated plants. In addition, the cultivars TE-resistance was enforced by the rising levels of phenolic compounds as well as the antioxidant enzymatic activities, which proportionally increased with contamination. However, slight variations were noted between the three cultivars in all the studied parameters, in which differences were mainly observed between the plants cultivated in the contaminated and uncontaminated soils. The various laboratory analyses were performed on samples collected during the first growing season. However, it might be useful to pursue the changes in cultivar reactions in response to TE contamination during further growing seasons. Moreover, the current study was carried out in pots with a specific quantity of soil, which might be considered a forcing environment. Therefore, further in situ experiments might be useful to validate the results obtained and specify the impact of TE contamination on miscanthus plants.

## Figures and Tables

**Table 1:** Particle size distribution, pH, Cd, Pb and Zn concentrations of the studied soils representing gradient TE concentration (MC-control, M200, M500, M750 and M900). Values are presented as means ± SD.

	MC	M200	M500	M750	M900
Clay (%)	20.8 ± 1.2	17.8 ± 1.5	30.6 ± 2.7	19.2 ± 1.2	16.4 ± 1.1
Silt (%)	69.5 ± 3.5	57.3 ± 3.6	49.7 ± 2.9	56 ± 3.7	59.2 ± 2.2
Sand (%)	9.7 ± 0.7	24.9	19.7 ± 1.3	24.8 ± 1.9	24.4 ± 2.1
pH	6.4 ± 0.1	7.1 ± 0.3	7.6 ± 0.2	7.3 ± 0.5	7.1 ± 0.2
Cd (mg kg^−1^)	0.3 ± 0.0	3.8 ± 0.2	9.0 ± 0.2	13.5 ± 0.3	16.0 ± 0.3
Pb (mg kg^−1^)	37.3 ± 1.3	260.3 ± 2.0	528.6 ± 5.3	747.1 ± 16.9	898.6 ± 16.3
Zn (mg kg^−1^)	54.6 ± 3.1	388.0 ± 14.8	537.0 ± 10.9	906.0 ± 16.8	1116.0 ± 1.7

**Table 2:** Leaf metal concentrations, number of tillers per pot, and stem diameter of the cultivars (B, U, and A) in soils with a gradient TE concentration (MC-control, M200, M500, M750, and M900) at the end of the first growing period.

		Cd (mg kg^−1^)	Pb (mg kg^−1^)	Zn (mg kg^−1^)	Number of Tillers	Stem Diameter (mm)
B	MC	<LD *	<LD *	32.2 + 2.8 fg	8.0 + 1.0 b	8.2 + 0.6 a
	M200	0.5 + 0.0 e	<LD *	60.5 + 2.6 bcd	4.0 + 1.6 bc	7.1 + 0.8 ab
	M500	1.0 + 0.1 d	<LD *	59.7 + 7.1 cd	5.0 + 1.6 bc	5.1 + 0.7 c
	M750	1.8 + 0.3 c	<LD *	72.9 + 8.5 abc	4.0 + 0.9 bc	5.3 + 0.6 bc
	M900	2.2 + 0.1 ab	<LD *	74.0 + 8.9 a	4.0 + 0.4 bc	4.9 + 0.8 c
U	MC	<LD *	<LD *	21.6 + 2.1 g	14.0 + 1.6 a	6.1 + 0.4 abc
	M200	0.5 + 0.1 e	<LD *	54.7 + 5.7 d	7.0 + 1.2 b	6.2 + 0.9 abc
	M500	0.9 + 0.1 d	<LD *	52.1 + 7.2 de	8.0 + 1.3 b	5.7 + 0.6 bc
	M750	2.1 + 0.3 abc	<LD *	64.8 + 5.5 abcd	5.0 + 0.8 bc	6.1 + 1.0 abc
	M900	2.3 + 0.2 a	<LD *	68.8 + 5.7 abc	5.0 + 1.6 bc	6.2 + 0.3 abc
A	MC	<LD *	<LD *	39.0 + 1.7 ef	8.0 + 1.7 b	7.4 + 1.2 ab
	M200	0.5 + 0.0 e	<LD *	60.0 + 5.8 cd	4.0 + 1.1 bc	5.9 + 0.7 abc
	M500	1.1 + 0.1 d	<LD *	62.6 + 3.3 abcd	4.0 + 1.0 bc	7.1 + 0.3 ab
	M750	1.9 + 0.1 bc	<LD *	71.2 + 5.7 abc	4.0 + 1.0 bc	5.7 + 0.5 bc
	M900	2.2 + 0.1 ab	<LD *	73.5 + 6.6 ab	4.0 + 0.7 bc	5.2 + 0.7 bc

Values are presented as means ± SD. Different letters refer to significant differences between plants (Turkey HSD test, *n* = 5, *p* ≤ 0.05). *: Limit of detection (Cd: 0.4 mg kg-1, Pb: 4.3 mg kg-1).

## Data Availability

Data sharing not applicable.

## Supplementary Materials

The following are available online at https://www.mdpi.com/article/10.3390/plants10102035/s1, Figure S1: Monthly average of atmospheric growth conditions impacting the miscanthus growth experiment (average temperature, precipitation, day sunlight and atmospheric pressure).
