# Supplementary material for: Response of Three Miscanthus × giganteus Cultivars to Toxic Elements Stress: Part 1, Plant Defence Mechanisms
Source: Plants (Basel). 2021 Sep 28;10(10):2035. doi: 10.3390/plants10102035 (PMC8538925; doi:10.3390/plants10102035)
Supplement: Supplementary file 1 [file plants-10-02035-s001.zip › plants-1399448-supplementary.pdf]

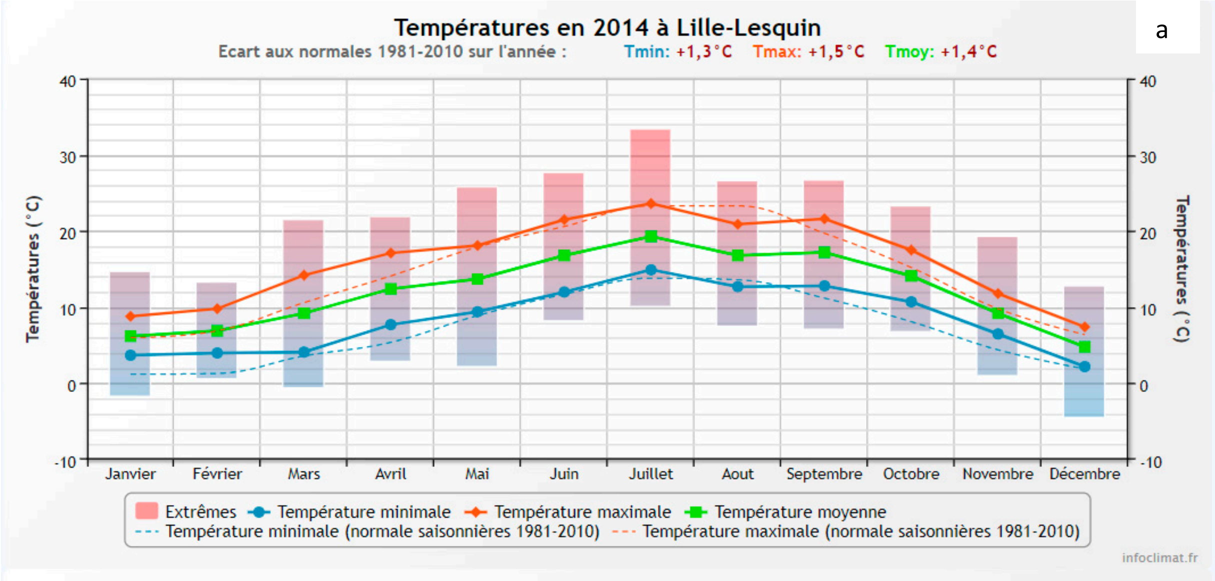
Températures en 2014 à Lille-Lesquin
Ecart aux normales 1981-2010 sur l'année : Tmin: +1,3°C Tmax: +1,5°C Tmoy: +1,4°C
a
Températures (°C)
Janvier Février Mars Avril Mai Juin Juillet Aout Septembre Octobre Novembre Décembre
Extrêmes
Température minimale
Température maximale
Température moyenne
Température minimale (normale saisonnières 1981-2010)
Température maximale (normale saisonnières 1981-2010)
infoclimat.fr

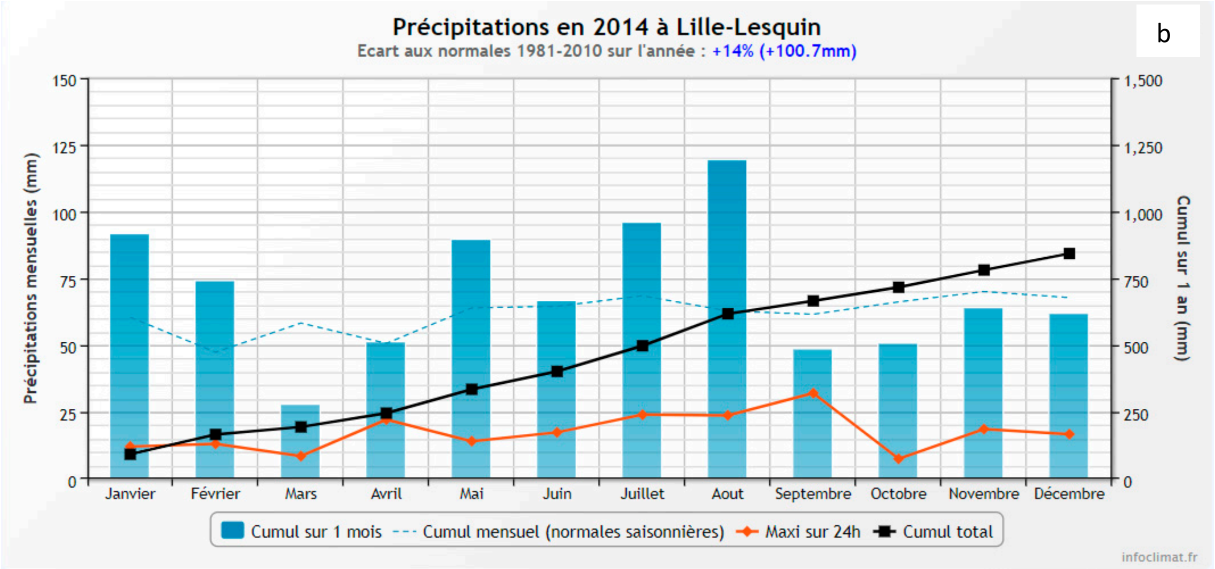
Précipitations en 2014 à Lille-Lesquin
Ecart aux normales 1981-2010 sur l'année : +14% (+100.7mm)
b
Précipitations mensuelles (mm)
Cumul sur 1 an (mm)
Janvier Février Mars Avril Mai Juin Juillet Aout Septembre Octobre Novembre Décembre
Cumul sur 1 mois
Cumul mensuel (normales saisonnières)
Maxi sur 24h
Cumul total
infoclimat.fr

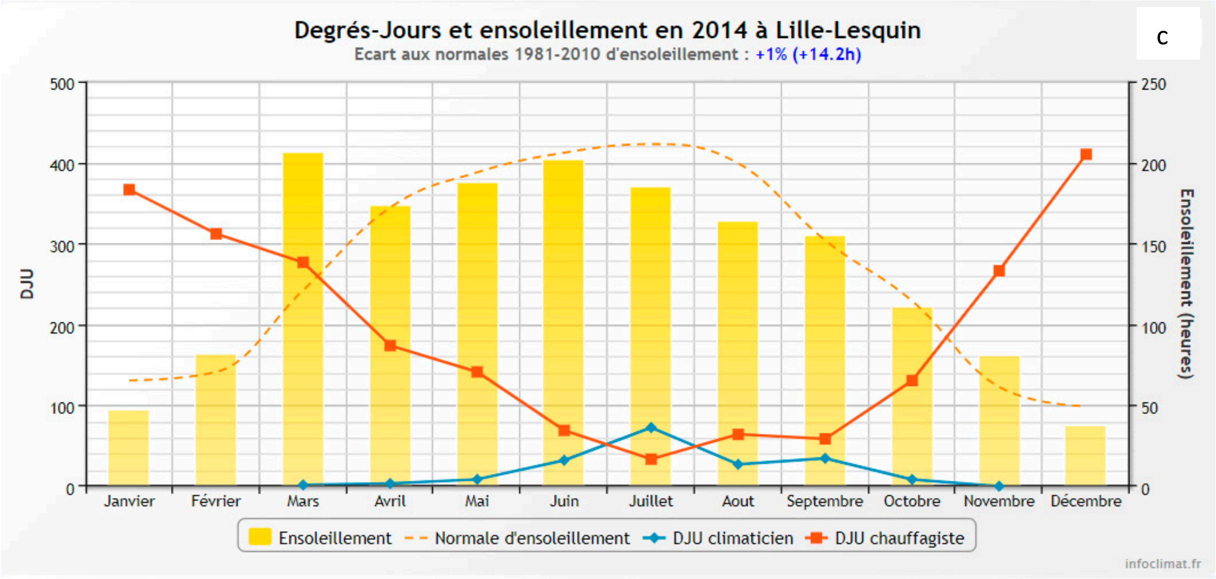

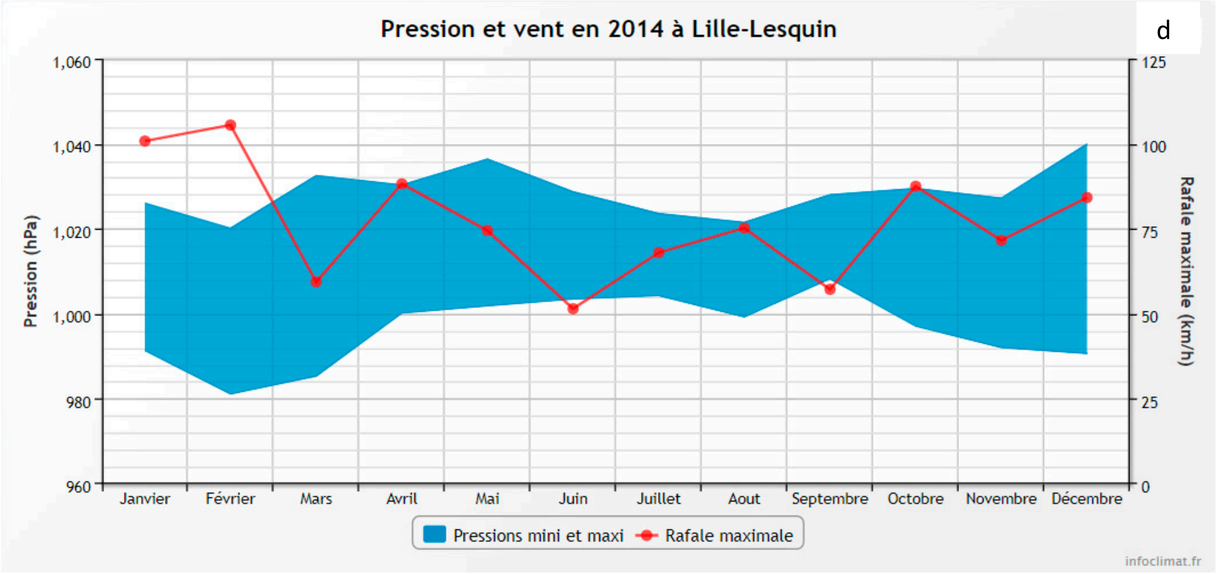

Figure S1 (a–d). Monthly average of atmospheric growth conditions impacting the miscanthus growth experiment (average temperature, precipitation, day sunlight and atmospheric pressure). Further details could be found on: https://www.infoclimat.fr/climatologie/annee/2014/lille-lesquin/valeurs/07015.html.
